# The mechanism of hamstring injuries – a systematic review

**DOI:** 10.1186/s12891-020-03658-8

**Published:** 2020-09-29

**Authors:** Adam Danielsson, Alexandra Horvath, Carl Senorski, Eduard Alentorn-Geli, William E. Garrett, Ramón Cugat, Kristian Samuelsson, Eric Hamrin Senorski

**Affiliations:** 1 Department of Orthopaedics, Sahlgrenska University Hospital, Mölndal, Sweden; 2 Department of Orthopaedics, Institute of Clinical Sciences, Sahlgrenska Academy, University of Gothenburg, Göteborgsvägen 31, SE-431 80 Mölndal, Gothenburg, Sweden; 3 Department of Internal Medicine and Clinical Nutrition, Institution of Medicine, Sahlgrenska Academy, University of Gothenburg, Gothenburg, Sweden; 4 Instituto Cugat, Barcelona, Spain; 5 Mutualidad Catalana de Futbolistas, Federación Española de Fútbol, Barcelona, Spain; 6 Fundación García-Cugat, Barcelona, Spain; 7 Duke Sports Sciences Institute, Duke University, Durham, North Carolina USA; 8 Department of Health and Rehabilitation, Institute of Neuroscience and Physiology, Sahlgrenska Academy, University of Gothenburg, Gothenburg, Sweden

**Keywords:** Running, Sprinting, Biomechanics, Strength, Muscle injury

## Abstract

**Background:**

Injuries to the hamstring muscles are among the most common in sports and account for significant time loss. Despite being so common, the injury mechanism of hamstring injuries remains to be determined.

**Purpose:**

To investigate the hamstring injury mechanism by conducting a systematic review.

**Study design:**

A systematic review following the PRISMA statement.

**Methods:**

A systematic search was conducted using PubMed, EMBASE and the Cochrane Library. Studies 1) written in English and 2) deciding on the mechanism of hamstring injury were eligible for inclusion. Literature reviews, systematic reviews, meta-analyses, conference abstracts, book chapters and editorials were excluded, as well as studies where the full text could not be obtained.

**Results:**

Twenty-six of 2372 screened original studies were included and stratified to the mechanism or methods used to determine hamstring injury: *stretch-related injuries, kinematic analysis, electromyography-based kinematic analysis and strength-related injuries*. All studies that reported the stretch-type injury mechanism concluded that injury occurs due to extensive hip flexion with a hyperextended knee. The vast majority of studies on injuries during running proposed that these injuries occur during the late swing phase of the running gait cycle.

**Conclusion:**

A stretch-type injury to the hamstrings is caused by extensive hip flexion with an extended knee. Hamstring injuries during sprinting are most likely to occur due to excessive muscle strain caused by eccentric contraction during the late swing phase of the running gait cycle.

**Level of evidence:**

Level IV

## Background

Hamstring injuries are common in several sports, with an overall incidence of 1.2–4 injuries per 1000 h of athlete exposure [1–3]. In athletics and Gaelic football, they account for 17–21% of total injuries [3, 4] and it is suggested that approximately 22% of all football players sustain a hamstring injury each season [1]. Hamstring injuries result in an average time loss of 24 days [5] and, result in high cost for professional athletes and teams [6]. Furthermore, dancers exhibit a high incidence of muscle injuries [7]. The relevance of hamstring injuries in sports is therefore paramount.

**Fig. 1:**
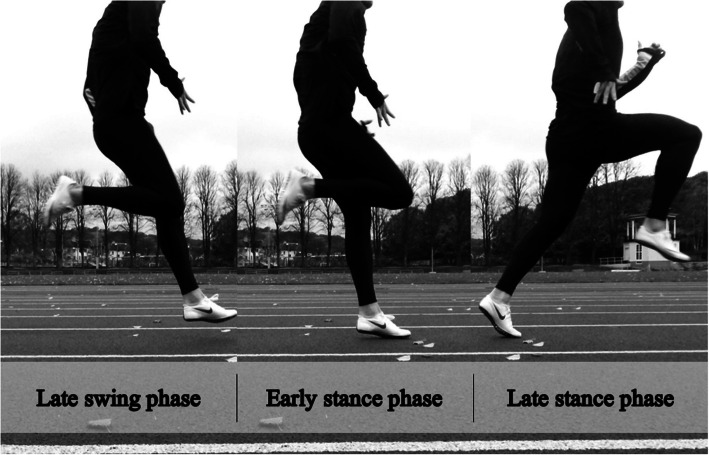
The running gait cycle

A growing body of research has focused on hamstring injuries, specifically to identify risk factors [8–10] and to develop prevention and rehabilitation programmes [11–15]. However, there is no consensus on hamstring injury mechanism. Askling et al. [16] proposed two scenarios in which a hamstring injury may occur; during either high-speed running, or stretching movements [16]. The high-speed running type of injury typically affects the long head of the biceps femoris (BFlh) and has a shorter recovery time than the stretching type of injury, which commonly affects the semimembranosus (SM) [17–19]. The running type of injury is the most frequent [20, 21] and, in Australian football, 81% of hamstring injuries occur during sprinting, while kicking (stretching type) accounts for 19% of injuries [2]. In the literature, there are two theories on the mechanism of hamstring injuries sustained during running. One is based on the findings of Garret and Lieber et al. [22, 23], who believed that the hamstring is most susceptible to injury during active lengthening, typically observed during the late swing phase of the running gait cycle (Fig. 1) [24]. As a result, preventive studies have focused on eccentric strengthening, with, for example, the Nordic hamstring exercise, which is associated with a significantly lower injury incidence [25–27]. Mann et al. [28], however, proposed that hamstring injury occurs during the initial stance phase because of the large forces in opposing directions as the body is propelled forward over the touchdown point (Fig. 1). By defining the mechanism of injury, new preventive strategies can hopefully be created to help reduce the number of hamstring injuries and re-injuries among athletes and patients. The aim of this study was to investigate the hamstring injury mechanism in a systematic review.

## Methods

The methodology of this study was reported following the Preferred Reporting Items for Systematic Reviews and Meta-Analyses (PRISMA) statement [29].

### Eligibility criteria

All the original studies that investigated the mechanism of hamstring injury or the biomechanical properties of the hamstrings were evaluated for eligibility. Hamstring injury was defined as a strain injury to the hamstring muscle group. Therefore, hamstring injuries with avulsion fractures were not considered for this systematic review. Studies were included if 1) they were written in English and; 2) conclusions were extrapolated on the mechanisms of hamstring injury. Literature reviews, systematic reviews, meta-analyses, conference abstracts, chapters from text-books and editorials were excluded, as well as studies where the full text could not be obtained.

### Information sources and search

#### Electronic search

A systematic electronic literature search was conducted on 21 February 2017 using the PubMed (first available date), EMBASE (starting in 1974) and the Cochrane Library (first available date) databases by an expert in electronic searching. An updated search was performed on 30 May 2018 for the PubMed and Cochrane, while an EMBASE search was updated on 7 June 2018. A third search was carried out on 10 July 2019. For all databases, a similar search strategy was used, where the only differences were due to database configuration. The search strategies used a combination of Medical Subject Heading (MeSH) terms and “title/abstract” search. The search strategy consisted of “hamstring *AND* injury *NOT* anterior cruciate ligament”, including synonyms (Tables 5, 6, 7 in Appendix).

#### Other search methods

The reference lists of all studies read in full text were screened for potential studies not previously identified.

### Data collection and analysis

#### Study selection

All titles and abstracts were read and studies of potential interest were reviewed in full text independently by two authors (Author 1 and Author 2) to decide on inclusion or exclusion. Disagreements were resolved through discussion with senior authors (Author 7 and Author 8).

#### Data collection process

The data extraction process was performed in duplicate (Author 1 and Author 2) using a piloted form of a Microsoft Excel (Microsoft, USA) spreadsheet and the following parameters were retrieved; author, year of publication, title, journal, number of study subjects, information on study subjects (age, sex) purpose, a detailed description of the methods used to assess injury mechanism (including important details such as the use of a treadmill or track, surface or needle electrodes, sampling rate if performing a video analysis, the use of reflective markers and/or force plates to measure ground reaction force), a summary of the results and the authors’ conclusions.

#### Data synthesis

The data synthesis was performed with a qualitative approach by gathering the authors’ results and conclusions, thereby excluding studies in which the hypothesised, suggested hamstring injury mechanism was not presented. Groups were created during the review process based on the common study methods used and different injury mechanisms reported. These groups are presented as *stretch-related injuries, kinematic analysis, electromyograph-based kinematic analysis and strength-related injuries* respectively.

### Quality appraisal of included studies

The included studies were evaluated for their reporting quality using the Downs and Black Checklist [30] comprising 27 items. Ten of the items refer to the reporting of study results, three items refer to external validity, 13 items to internal validity and one item to power calculation. Since none of the included studies was interventional and only one study had comparative groups, a total of 16 items were used, while 11 were excluded from the qualitative analysis (items 4–5, 8, 13–15, 19, 21–24). Of the 16 items used, seven examined the reporting of information, two examined external validity, six investigated internal validity and one item was related to power calculation. Each item can be answered yes (1 point), no (0 points) and unable to determine (0 points), except item 27, which may yield up to five points depending on the power calculation. The maximum score on the modified Down and Blacks Checklist is 20. However, not all of the 16 included items were applicable to each individual study, as study methodologies differed. Two authors (Author 1 and Author 2) independently performed the quality appraisal and differences were resolved with discussion (Table 8 in Appendix).

## Results

### Study selection

**Fig. 2:**
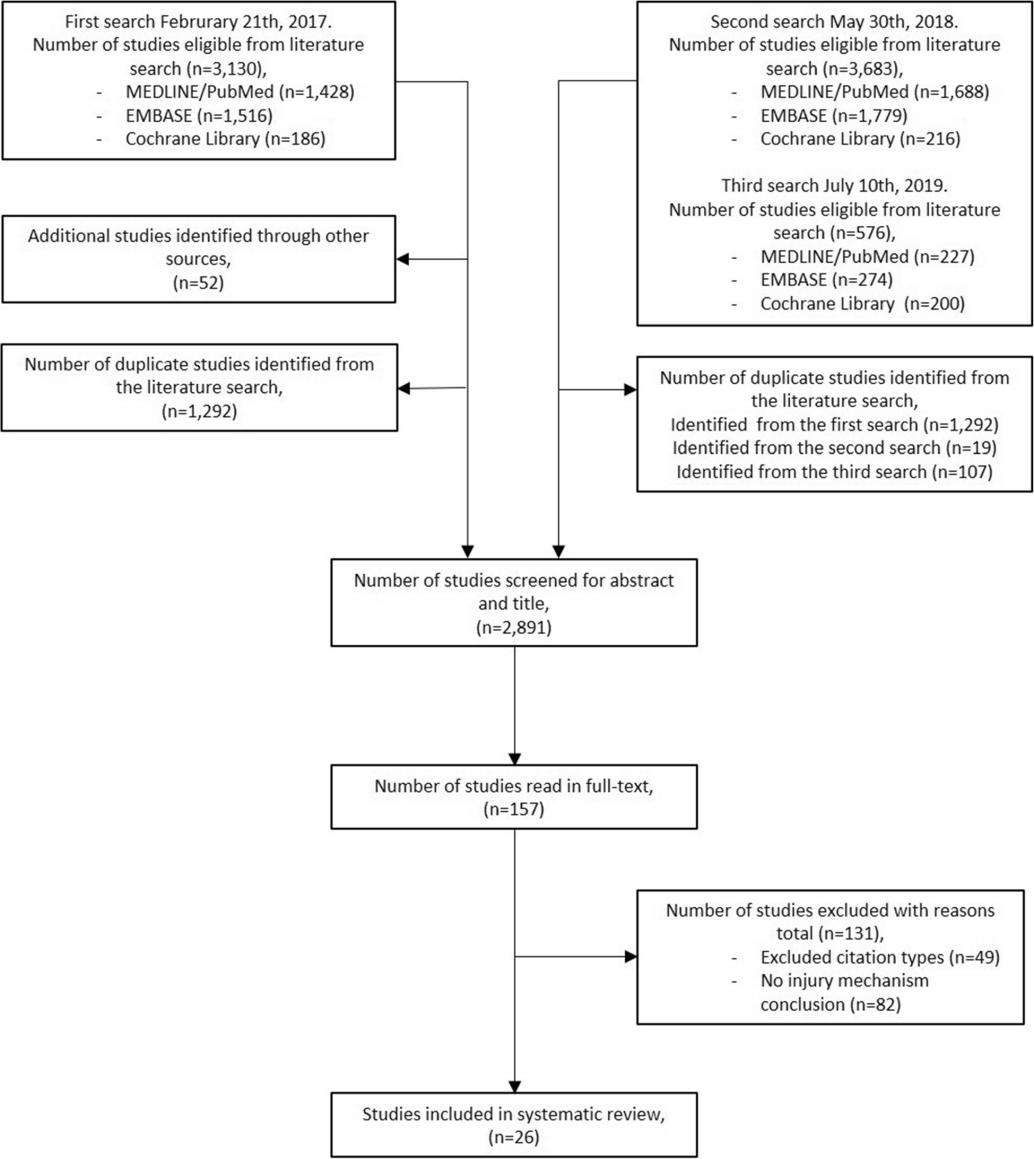
The inclusion and exclusion of studies

The database search identified 318 studies from the Cochrane Library, 2053 from EMBASE and 1893 from PubMed, giving a total of 4264 studies. After the removal of the 1423 duplicates, the remaining 2841 studies were screened by abstract and title. Eligible studies underwent full text assessment and 21 studies were included in the final systematic review. During the full text assessment, 52 previously unidentified studies were identified from the reference lists (Fig. 2), of which five studies were eligible for inclusion [19, 28, 31–33].

### Risk of bias assessment

The quality appraisal with a modified version of the Downs and Black Checklist [30] resulted in a median (range) score of 8 (7–14) points of 20 possible. See Table 1 for full results.

**Table 1:** Scoring from the modified Downs and Black Checklist assessing risk of bias. Certain items were not applicable to all studies

Downs and Black Checklist item																	
Authors	1 *Hypothesis described*	2 *Main outcome described*	3 *Patient characteristics described*	6 *Main finding described*	7 *Estimates of outcome variability*	9 *Characteristics of patient lost to follow-up*	10 *Actual probability values*	11 *Subjects asked to participate representative*	12 *Subjects prepared to participate representative*	16 *Results based on data dredging made clear*	17 *Different length of follow-up adjusted*	18 *Appropriate statistics*	20 *Outcome valid and reliable*	25 *Adjustment for confounders*	26 *Loss to follow-up taken into account*	27 *Sufficient power*	Sum
Askling et al. [19]	1	1	1	1	1	1	0	0	0	1	1	1	1	N/A	1	0	11
Askling et al. [31]	1	1	1	1	1	1	N/A	0	0	1	N/A	1	1	N/A	1	0	10
Chumanov et al. [34]	1	1	1	1	1	N/A	1	N/A	N/A	1	N/A	1	1	N/A	N/A	0	9
Fiorentino et al. [32]	1	1	1	1	1	N/A	N/A	0	0	1	N/A	1	1	N/A	N/A	N/A	8
Hanley et al. [35]	1	1	1	1	1	N/A	1	0	0	1	N/A	1	1	N/A	N/A	0	9
Hanley et al. [36]	1	1	1	1	1	N/A	1	0	0	1	N/A	1	1	N/A	N/A	0	9
Heiderscheit et al. [37]	1	1	1	1	1	N/A	N/A	N/A	N/A	1	N/A	1	1	N/A	N/A	N/A	8
Higashihara et al. [38]	1	1	1	1	1	N/A	0	0	0	1	N/A	1	1	N/A	N/A	0	8
Higashihara et al. [39]	1	1	1	1	1	N/A	0	0	0	1	N/A	1	1	N/A	N/A	0	8
Jones et al. [40]	1	1	1	1	1	N/A	1	0	0	1	N/A	1	1	N/A	N/A	0	9
Mann et al. [28]	0	1	1	1	1	N/A	1	0	0	1	N/A	1	1	N/A	N/A	N/A	8
Montgomery III et al. [33]	1	1	1	1	1	N/A	0	0	0	1	N/A	1	1	N/A	N/A	0	8
Ono et al. [41]	1	1	1	1	1	N/A	N/A	0	0	1	N/A	1	1	N/A	N/A	0	8
Padulo et al. [42]	1	1	1	1	1	N/A	0	0	0	1	N/A	1	1	N/A	N/A	0	8
Prior et al. [43]	1	1	1	1	1	N/A	1	0	0	1	N/A	1	1	N/A	N/A	5	14
Ruan et al. [44]	1	1	1	1	1	N/A	0	0	0	1	N/A	1	1	N/A	N/A	0	8
Sallay et al. [45]	1	1	1	1	1	N/A	N/A	0	0	1	1	N/A	1	N/A	N/A	N/A	8
Schache et al. [46]	1	1	1	1	1	N/A	N/A	0	0	1	N/A	1	1	N/A	N/A	N/A	8
Schache et al. [47]	1	1	1	1	1	N/A	N/A	0	0	1	N/A	1	1	N/A	N/A	N/A	8
Schache et al. [48]	1	1	1	1	1	N/A	N/A	N/A	N/A	1	N/A	1	1	N/A	N/A	N/A	8
Schuermans et al. [49]	1	1	1	1	1	N/A	1	0	0	1	N/A	1	1	1	N/A	0	10
Schuermans et al. [50]	1	1	1	1	1	1	1	0	0	1	1	1	1	0	0	0	11
Sun et al. [51]	1	1	1	1	1	N/A	N/A	0	0	1	N/A	1	1	N/A	N/A	N/A	8
Thelen et al. [52]	1	1	0	1	1	N/A	0	0	0	1	N/A	1	1	N/A	N/A	0	7
Wan et al. [53]	1	1	1	1	1	N/A	1	0	0	1	N/A	1	1	N/A	N/A	0	9
Yu et al. [54]	1	1	1	1	1	N/A	1	0	0	1	N/A	1	1	N/A	N/A	0	9

*N/A* Not applicable

### Characteristics of included studies

Of the 26 studies included, three investigated stretch-type hamstring injuries [19, 31, 45], 10 performed a kinematic analysis [28, 32, 35, 37, 39, 46, 47, 51–53], 10 additional studies performed a kinematic analysis combined with an electromyographic (EMG) analysis [33, 34, 36, 38, 41–44, 48, 54] and three analysed muscle strength [40, 49, 50]. The number of participants in the included studies ranged from one to 54 (total of 444 participants; some individuals included in more than one study) with an age range of 16–53 years.

Six studies analysed actual hamstring injuries [19, 31, 37, 45–47], one study compared previously injured and uninjured individuals [49], while 19 studies performed the analyses on uninjured individuals and estimated the hamstring injury mechanism [28, 32–36, 38–44, 48, 50–54]. A summary of the suggested hamstring injury mechanisms is presented in Table 2 and a comprehensive summary of the included studies can be found in Table 9 in Appendix.

**Table 2:** Summary of the suggested hamstring injury mechanisms and most injury-prone phase stratified by results and method used to investigate injury mechanism

Results according to injury mechanism and study method	Number of studies
**Stretch-type injury**	3
Hyperextension [19, 31, 45]	3
**Kinematics**	10
Swing phase [32, 35, 37, 46, 47, 52, 53]	7
Stance phase [28, 39]	2
Both phases [51]	1
**Kinematics with electromyographic analysis**	9
Swing phase [33, 34, 36, 38, 48]	5
Stance phase [41]	1
Two phases [44, 54]	2
Other [42, 43]	2
**Strength**	3
Fatigue [40, 49]	2
Asymmetrical activation [50]	1

### Stretch-related hamstring injuries

Three studies investigated hamstring injuries in dancers and water skiers and scored a median (range) of 10 points (8–11) out of 20 possible on the modified Downs and Black Checklist. The study populations ranged from 12 to 30 subjects aged 16–53 years who participated in interviews and clinical and magnetic resonance imaging (MRI)) examinations to determine the hamstring injury mechanism. All three studies reported that hamstring injuries occurred due to extensive hip flexion with a hyperextended knee [19, 31, 45]. In one study of dancers, the quadratus femoris and adductor magnus were injured simultaneously with the hamstrings [19].

### Hamstring injury mechanism from kinematic analysis

Ten studies investigated the hamstrings through a kinematic analysis of study subjects aged 16–31 years with a median (range) score of 8 (7–9) of 20 possible on the modified Downs and Black Checklist. Nine of these studies were conducted on runners [28, 32, 37, 39, 46, 47, 51–53] and one on race walkers [35]. with study populations ranging from one to 20 participants. High-speed cameras and skin-placed markers on anatomic landmarks were most commonly used to study the injuries while the subjects ran on a treadmill or track. In four studies, a force plate was added to obtain additional information [35, 46, 47, 51]. One study measured BFlh dimensions using MRI images which were subsequently used in a simulation of hamstring injury mechanics [32]. Three studies were able to record a hamstring injury in real time [37, 46, 47]. However, two of these studies based their conclusions on data from the same study subject [46, 47]. Seven studies made estimations of where the hamstrings were at highest risk of injury [28, 32, 35, 39, 51–53].

Two studies reported that hamstring injuries occur during the early stance phase [28, 39], while running with a forward trunk lean [39]. In contrast, seven studies concluded that hamstring injuries occur during the swing phase [32, 35, 37, 46, 47, 52, 53] and one study concluded that both phases exhibit a risk of injury [51]. It was proposed that the late or terminal swing phase placed the hamstring muscles at the highest risk of injury (Table 3).

**Table 3:** Methodological characteristics of the kinematic studies

Authors	Study population	Data collection	Surface	Force plates	Injured athlete	Parameter used to draw conclusion	Conclusion
Fiorentino et al. [32]	14 track and field athletes	Computational model based on hamstring dimensions	N/A	No	No	Calculated local fibre strain	Late swing phase
Hanley et al. [35]	17 race walkers	High-speed camera	Track	Yes	No	Energy absorption	Swing phase
Heiderscheit et al. [37]	1 runner	Reflective markers and high-speed camera	Treadmill	No	Yes	Earliest sign of reaction to injury, including neuromuscular latencies	Late swing phase
Higashihara et al. [39]	8 runners	Reflective markers and high-speed camera	Track	No	No	Muscle length	Stance phase
Mann et al. [28]	15 runners	Reflective markers and high-speed camera	Track	No	No	Passive torques	Early stance phase
Schache et al. [46]	1 runner	Reflective markers and high-speed camera	Track	Yes	Yes	Hamstring length, force, velocity and negative work	Terminal swing phase
Schache et al. [47]	1 runner	Reflective markers and high-speed camera	Track	Yes	Yes	Earliest sign of reaction to injury, including neuromuscular latencies	Terminal swing phase
Sun et al. [51]	8 runners	Reflective markers and high-speed camera	Track	Yes	No	Passive torques	Late swing and early stance phase
Thelen et al. [52]	14 runners	Reflective markers and high-speed camera	Treadmill	No	No	Muscle length	Late swing phase
Wan et al. [53]	20 runners	Reflective markers and high-speed camera, isometric strength and flexibility	Track	No	No	Peak muscle strain	Late swing phase

*N/A* Not applicable

### Hamstring injury mechanism from kinematic and electromyographic analysis

Ten studies performed EMG-based kinematic analysis [33, 34, 36, 38, 41–44, 48, 54] measured with either surface or needle electrodes [33] and, in some cases, with additional force plates [36, 41, 48]. The modified Downs and Black Checklist yielded a total median (range) score of 8 (8–14) of 20 possible for these studies. Seven studies analysed runners [33, 34, 38, 41, 44, 48, 54], one study used race walkers [36], one evaluated volleyball players performing different jumping tasks [42] and one study compared muscle activity while standing on one leg with different trunk and pelvic positions in healthy volunteers [43]. The studies included recreational and high-level athletes with an age range of 18–53 years and consisted of seven to 30 individuals.

One study concluded that the risk of hamstring injury is greatest during the early stance phase [41], while five studies reported that injury occurred during the swing phase [33, 34, 36, 38, 48]. One study suggested that hamstring injury may occur during either the early stance phase or late swing phase [44], while another study reported that injury could occur during both the late stance and late swing phase (Fig. 1) [54].

One study reported that anterior trunk sway and contralateral pelvic drop while standing on one leg increased the load on the hamstrings [43], while another study reported that the hamstrings are at risk of injury during concentric, braking movements [42]. All conclusions were based on estimations of when the highest risk of hamstring injury occurs, i.e. no study included an actual hamstring injury (Table 4).

**Table 4:** Methodological characteristics of the kinematic studies with concomitant electromyographic analyses

Authors	Study population	Data collection	Surface	Force plates	Injured athlete	Parameter used to draw conclusion	Conclusion
Chumanov et al. [34]	12 runners	Surface electrodes, reflective markers and high-speed cameras	Tread-mill	No	No	Eccentric contraction	Late swing phase
Hanley et al. [36]	20 race walkers	High-speed cameras and surface electrodes	Track	Yes	No	Energy absorption	Swing phase
Higashihara et al. [38]	13 runners	Surface electrodes, reflective markers and high-speed cameras	Track	No	No	Musculotendon length and EMG activity	Late swing phase
Montgomery III et al. [33]	30 runners	Needle electrodes and high-speed camera	Track	No	No	Eccentric contraction	Swing phase
Ono et al. [41]	12 runners	Surface electrodes, reflective markers and high-speed cameras	Track	Yes	No	Tensile force index = length x EMG activity	Early stance phase
Padulo et al. [42]	12 volleyball players	Surface electrodes and high-speed cameras during jumping exercises	N/A	No	No	Neuromuscular activity	Pure concentric moves are more injury prone than stretch-shortening moves
Prior et al. [43]	22 asymptomatic males	Surface electrodes, reflective markers and high-speed cameras	N/A	No	No	Neuromuscular activity	Anterior trunk sway and lateral pelvic drop increases hamstring loading and may affect injury risk
Ruan et al. [44]	12 healthy female sprinters	Surface electrodes, reflective markers and high-speed cameras	Track	Yes	No	Tendon stiffness, tension-length curve and GRF	Late swing and early stance phase
Schache et al. [48]	7 runners	Surface electrodes, reflective markers and high-speed cameras	Track	Yes	No	Lengthening of the hamstrings, peak force and the amount of negative work performed	Terminal swing phase
Yu et al. [54]	20 runners	Surface electrodes, reflective markers and high-speed cameras	Track	No	No	Eccentric contraction	Late swing and late stance phase

*EMG* Electromyography, *GRF* Ground reaction force, *N/A* Not applicable

### Strength-related hamstring injuries

Three studies investigated hamstring strength in football players aged 18–35 years [40, 49, 50] and scored a median (range) value of 10 points (9–11) of 20 possible on the modified Downs and Black Checklist. One study measured seated isokinetic strength in 20 football players prior to, during and after an exercise protocol set to simulate the muscle fatigue induced by a football game [40]. It was reported that hamstring injury was caused by lower eccentric strength due to fatigue [40]. Two studies used muscle functional magnetic resonance imaging (mfMRI) to compare metabolic activity before and after an eccentric hamstring exercise in previously uninjured and injured football players [49, 50]. One study reported that previously injured athletes had lower eccentric endurance of the hamstrings compared with uninjured athletes. It was proposed that the inferior hamstring endurance was a result of less economic muscle activation which may constitute a risk for injury [49]. One study performed an MRI analysis before and after an eccentric hamstring exercise and registered hamstring injuries for the following 1.5 seasons [50]. The results indicated that a greater contribution from the biceps femoris compared with the semitendinosus (ST) during an eccentric hamstring exercise correlates with first-time hamstring injuries. Re-injuries were associated with lower eccentric hamstring endurance [50].

## Discussion

Across studies that investigated runners, the most commonly suggested injury mechanism was eccentric strain during the late swing phase of the running gait cycle. In a sub-group of hamstring injuries, the reviewed studies reported that the mechanism of hamstring injuries includes a simultaneous hip flexion and knee extension.

### Stretch-related hamstring injuries

All the studies [19, 31, 45] of stretch-type injuries concluded that injuries occur due to extensive hip flexion with simultaneous knee extension. The study methods were similar, with a qualitative interview on the injury situation as the main source of information. In Australian football, a total of 19% of hamstring injuries occur during kicking [2], which is a typical stretch-type hamstring injury, given that the end of a kick exhibits both a flexed hip and extended knee position. In addition, Worth [55] suggested that trying to pick up a ball from the ground while running at full speed is the most common hamstring injury situation in Australian football. Picking up something from the ground may exhibit the same traits as the stretch-type hamstring injuries, further supporting this theory [55]. Notably, these studies analysed patients who had sustained hamstring injuries. However, since none of the hamstring injuries was observed by the researchers, the injury situations were recalled by the patient, thereby entailing a risk of bias. The findings relating to stretch-type hamstring injury should therefore be interpreted with caution.

### Hamstring injuries during running

The majority of studies of hamstring injuries during running reported that the hamstrings are most prone to injury during the late swing phase as a result of eccentric loading. However, some studies reported that the hamstrings are most likely to be injured during the stance phase. It is pivotal to acknowledge that, in cases in which an accidental hamstring injury was recorded in real time, the authors concluded that the injury occurred during the late swing phase [37, 46, 47]. This information was concluded through the earliest sign of injury including neuromuscular latencies [37, 47] as well as examining hamstring length, force, velocity and negative work [46]. This is in line with the findings of a recent literature review which suggests that hamstring injury during the late swing phase occurs due to high levels of muscle excitation and muscle strain [56]. Interestingly, Mendiguchia et al. [57] were able to record a hamstring injury and, while no injury mechanism was reported, the authors stated that the injury occurred when the subject ran with an “abnormal increase in power compared with velocity qualities” [57].

One study concluded that a hamstring injury is most likely to occur during the stance phase when comparing a normal running technique with a technique in which the subjects run with a forward trunk lean [39]. These results are in line with the findings of Prior et al. [43], who reported that an anterior trunk sway during single leg stance, similar to positions which occur in pivoting sports, increased hamstring strain [43]. However, strain on the hamstring muscles and injury conditions during running with a forward trunk lean may differ from a normal running technique as the forwards trunk lean elongates the hamstring muscle causing more strain. Interestingly, a forward trunk lean had the greatest impact during the stance phase with the knee fully extended, similar to the stretch-type injury mechanism. The forward trunk lean can be caused by poor activation and control of the muscles of the core and hip, thereby increasing the strain and injury risk of the hamstrings [58–61]. For this reason, an in-depth knowledge of this type of injury is imperative and could be implemented in hamstring injury prevention and rehabilitation programmes, focusing on hip and core strengthening exercises in addition to traditional hamstring exercises.

Furthermore, static stretching may reduce both the ground reaction forces observed in the early stance phase and the strain on the BFlh during the late swing phase [44]. This results in subsequent reduced peak values of joint torque at the hip and knee and increased force productions of the biceps femoris at longer muscle lengths, which demonstrates that stretching may reduce the risk of hamstring injuries [44, 56]. These findings are of particular interest as preventive studies on the Nordic hamstring exercise which focuses on eccentric training have shown to reduce the risk of hamstring injuries [25–27]. The preventive effect of the Nordic hamstring exercise may be attributed to its ability to increase muscle fascicle length [62] as short hamstring fascicles are associated with an increased risk of a hamstring injury [63].

The results of a study of muscle activity during running and preventive exercises for the hamstrings suggested that the highest activity of the hamstrings occurs during the late swing phase [64], potentially associated with an increased risk of injury. On the other hand, Ono et al. [41] reported that, during the swing phase, the tensile forces in the ST exceed the forces in the BFlh, while the BFlh during the stance phase demonstrates higher forces. Since it is more common to injure the BFlh while running compared with the ST, the authors suggested that hamstring injury probably occurs during the stance phase [18]. In addition, the medial hamstrings are primarily loaded during the swing phase, where the lateral hamstrings are active throughout the entire gait cycle [65], which may help to explain why the ST is less injured, despite the high force [41].

In the light of these findings, several limitations need to be mentioned. There were only three case reports that studied recordings of a real-time hamstring injury [37, 46, 47] and the same study subject was used in two of the case reports [46, 47]. Furthermore, contextual conditions varied between studies, where, in some studies, the running analyses were performed on a treadmill [34, 37, 52] and had subjects running at a slow pace, which may not reflect the mechanism of hamstring injury. Since hamstring injuries commonly affect athletes playing various sports on grass fields, there is a lack of studies examining the injury mechanism in those conditions. The results in current literature may therefore prove difficult to apply to hamstring injuries sustained on grass. In addition, some studies performed a kinematic analysis without the use of an EMG which, it can be argued, only investigates hamstring lengthening and not active lengthening, i.e. eccentric contraction, as muscle activity is not measured.

In conclusion, hamstring injuries sustained while running or sprinting are estimated to occur during the late swing phase as a consequence of increased strain on the hamstring muscles. However, further research is needed to confirm these findings.

### Strength-related hamstring injuries

There are inconclusive results from retrospective studies of hamstring strength in relation to the mechanism of injury. Fatigue was reported to reduce eccentric hamstring strength, which was suggested to increase the risk of a hamstring injury [40], while lower hamstring strength endurance was associated with a hamstring re-injury [50]. One study compared muscle activity in athletes with previously injured and uninjured hamstrings and reported that the previously injured athletes had inferior hamstring activation, which contributes to lower hamstring strength [49]. These findings are most probably related to risk factors for suffering a subsequent injury, which may in turn help to improve rehabilitation, rather than being related to the mechanism of hamstring injury [1, 5, 66].

### Limitations

Most importantly, the majority of studies based their conclusions on estimations of the hamstring injury mechanism. Furthermore, the number of publications relating to the hamstring injury mechanism is limited and different methods have been used to assess the mechanism of injury. As a result, the included studies were allocated to groups defined by the study method and mechanism of injury. Each group included a limited number of studies with different methodological limitations which resulted in uncertainty about the results in this systematic review. In addition, a number of biomechanical studies were excluded, as no conclusions were drawn with regard to the hamstring injury mechanism. The extensive manual search of the reference lists of included studies helped to identify additional literature on the hamstring injury mechanism. However, the inclusion criterion of *“conclusions were extrapolated by the authors with regard to the mechanisms of hamstring injury”* may have introduced bias, as studies either estimated the mechanism of injury or retrospectively reviewed hamstring injuries and not an actual injury per se. Also, only studies written in English were set to be included but throughout the process of manually searching reference lists no studies were excluded for this reason.

The Downs and Black Checklist was deemed the most correct to determine the reporting quality of included studies, although it was not completely suited to the study designs included. The overall interpretation of reporting quality was low, with a risk of bias related primarily to study size and design, although there are no cut-offs or standardised methods for interpreting the modified version of the Downs and Black Checklist.

## Conclusion

A stretch-type injury to the hamstrings is caused by extensive hip flexion with an extended knee. Hamstring injuries during sprinting are most likely to occur due to excessive muscle strain caused by eccentric contraction during the late swing phase of the running gait cycle.

## Data Availability

All data generated or analysed during this study are included in this published article [and its supplementary information files].

## Appendix

**Table 5:** Search strategy PubMed

Database: PubMed		
Date: 2017-02-21		
Number of results: 1428 references		
Search	**Query**	**Items found**
#6	**Search #3 NOT #4 Filters: English**	**1428**
#5	Search #3 NOT #4	1480
#4	Search Anterior cruciate ligament[ti] OR patellar tendon[ti] OR ACL[ti] OR posterior cruciate ligament[ti]	13007
#3	Search #1 AND #2	2384
#2	Search “Wounds and Injuries”[Mesh:NoExp] OR “Athletic Injuries”[Mesh] OR “Leg Injuries”[Mesh:NoExp] OR “Sprains and Strains”[Mesh:NoExp] OR “Tendon Injuries”[Mesh:NoExp] OR “injuries”[Subheading] OR injury[tiab] OR injuries[tiab] OR tear[tiab] OR tears[tiab] OR rupture[tiab] OR strain[tiab] OR strains[tiab]	1483367
#1	Search “Hamstring Tendons”[Mesh] OR “Hamstring Muscles”[Mesh] OR hamstring[tiab] OR hamstrings[tiab]	6169
Database: PubMed		
Date: 2018-05-30		
Number of results: 1688 references		
Search	**Query**	**Items found**
#1	Search ((“Hamstring Tendons”[Mesh] OR “Hamstring Muscles”[Mesh] OR hamstring[tiab] OR hamstrings[tiab]) AND (“Wounds and Injuries”[Mesh:noexp] OR “Athletic Injuries”[Mesh] OR “Leg Injuries”[Mesh:noexp] OR “Sprains and Strains”[Mesh:noexp] OR “Tendon Injuries”[Mesh:noexp] OR “injuries”[Subheading] OR injury[tiab] OR injuries[tiab] OR tear[tiab] OR tears[tiab] OR rupture[tiab] OR strain[tiab] OR strains[tiab])) NOT (Anterior cruciate ligament[ti] OR patellar tendon[ti] OR ACL[ti] OR posterior cruciate ligament[ti]) Filters: English	**1688**
Database: PubMed		
Date: 2018-05-30		
Number of results: 1688 references		
Search	**Query**	**Items found**
#10	**Search #7 AND #8 Filters: English**	**200**
#9	Search #7 AND #8	205
#8	Search “2018/05/30”[crdt]: “2019/07/10”[crdt]	1454534
#7	Search #5 NOT #6	1960
#6	Search Anterior cruciate ligament[ti] OR patellar tendon[ti] OR ACL[ti] OR posterior cruciate ligament[ti]	15670
#5	Search #3 AND #4	3156
#4	Search “Wounds and Injuries”[Mesh:noexp] OR “Athletic Injuries”[Mesh] OR “Leg Injuries”[Mesh:noexp] OR “Sprains and Strains”[Mesh:noexp] OR “Tendon Injuries”[Mesh:noexp] OR “injuries”[Subheading] OR injury[tiab] OR injuries[tiab] OR tear[tiab] OR tears[tiab] OR rupture[tiab] OR strain[tiab] OR strains[tiab]	1687635
#3	Search “Hamstring Tendons”[Mesh] OR “Hamstring Muscles”[Mesh] OR hamstring[tiab] OR hamstrings[tiab]	7710

**Table 6:** Search Strategy EMBASE

Database: EMBASE 1974 to 2017 February 17		
Date: 2017-02-21		
Number of results: 1516 references		
**#**	**Search**	**Hits**
1	exp hamstring/	6601
2	(hamstring or hamstrings).ab,ti.	7417
3	1 or 2	8479
4	*injury/	109,627
5	muscle injury/	11,322
6	leg injury/	8177
7	exp tendon injury/	20,052
8	sport injury/	27,033
9	*musculoskeletal injury/ or sprain/	4077
10	4 or 5 or 6 or 7 or 8 or 9	169,425
11	(injury or injuries or tear or tears or rupture or strain or strains).ab,ti.	1,556,494
12	10 or 11	1,624,147
13	3 and 12	3061
14	(Anterior cruciate ligament or patellar tendon or ACL or posterior cruciate ligament).ti.	14,978
15	13 not 14	1954
16	**limit 15 to (English and (article or conference paper or note or “review”))**	**1516**
Database: EMBASE 1974 to 2018 June 7		
Date: 2018-06-07		
Number of results: 1779 references		
*#*	**Search**	**Hits**
1	exp hamstring/	3723
2	(hamstring or hamstrings).ab,ti.	8371
3	1 or 2	10,897
4	*injury/	63,184
5	muscle injury/	10,903
6	leg injury/	8268
7	exp tendon injury/	20,447
8	sport injury/	27,738
9	*musculoskeletal injury/ or sprain/	3474
10	4 or 5 or 6 or 7 or 8 or 9	128,054
11	(injury or injuries or tear or tears or rupture or strain or strains).ab,ti.	1,680,755
12	10 or 11	1,737,093
13	3 and 12	3656
14	(Anterior cruciate ligament or patellar tendon or ACL or posterior cruciate ligament).ti.	16,690
15	13 not 14	2324
16	**limit 15 to (English and (article or conference paper or note or “review”))**	**1779**
Database: EMBASE 1974 to 2019 July 9		
Date: 2019-07-10		
Number of results: 274 references		
*#*	**Search**	**Hits**
1	exp hamstring/	4984
2	(hamstring or hamstrings).ab,ti.	9410
3	1 or 2	12,461
4	*injury/	59,371
5	muscle injury/	11,874
6	leg injury/	7328
7	exp tendon injury/	21,448
8	sport injury/	28,438
9	*musculoskeletal injury/ or sprain/	3831
10	4 or 5 or 6 or 7 or 8 or 9	126,329
11	(injury or injuries or tear or tears or rupture or strain or strains).ab,ti.	1,771,861
12	10 or 11	1,824,435
13	3 and 12	4260
14	(Anterior cruciate ligament or patellar tendon or ACL or posterior cruciate ligament).ti.	18,599
15	13 not 14	2711
16	limit 15 to (english and (article or conference paper or note or “review”))	2038
17	**limit 16 to dc = 20,180,607–20,190,710**	**274**

“*” is part of the EMBASE database configuration

**Table 7:** Search strategy The Cochrane Library

Database: The Cochrane Library		
Date: 2017-02-21		
Number of results: 186 references		
*Cochrane reviews: 2*		
*Other reviews: 8*		
*Trials: 176*		
ID	**Search**	**Hits**
#1	hamstring or hamstrings:ti,ab,kw (Word variations have been searched)	1043
#2	MeSH descriptor: [Wounds and Injuries] this term only	1460
#3	MeSH descriptor: [Athletic Injuries] explode all trees	599
#4	MeSH descriptor: [Leg Injuries] explode all trees	3283
#5	MeSH descriptor: [Leg Injuries] this term only	177
#6	MeSH descriptor: [Sprains and Strains] this term only	326
#7	MeSH descriptor: [Tendon Injuries] this term only	239
#8	#2 or #3 or #4 or #5 or #6 or #7	5423
#9	injury or injuries or tear or tears or rupture or strain or strains:ti,ab,kw (Word variations have been searched)	42,988
#10	#8 or #9	44,566
#11	#1 and #10	406
#12	anterior cruciate ligament or “patellar tendon” or ACL or “posterior cruciate ligament”:ti (Word variations have been searched)	1551
#13	**#11 not #12**	**186**
Date: 2018-05-30		
Number of results: 216 references		
*Cochrane reviews: 2*		
*Other reviews: 8*		
*Trials: 206*		
ID	**Search**	**Hits**
#1	hamstring or hamstrings:ti,ab,kw (Word variations have been searched)	1202
#2	MeSH descriptor: [Wounds and Injuries] this term only	1535
#3	MeSH descriptor: [Athletic Injuries] explode all trees	664
#4	MeSH descriptor: [Leg Injuries] explode all trees	3803
#5	MeSH descriptor: [Leg Injuries] this term only	188
#6	MeSH descriptor: [Sprains and Strains] this term only	341
#7	MeSH descriptor: [Tendon Injuries] this term only	256
#8	#2 or #3 or #4 or #5 or #6 or #7	6043
#9	injury or injuries or tear or tears or rupture or strain or strains:ti,ab,kw (Word variations have been searched)	51,335
#10	#8 or #9	53,039
#11	#1 and #10	494
#12	anterior cruciate ligament or “patellar tendon” or ACL or “posterior cruciate ligament”:ti (Word variations have been searched)	1696
#13	**#11 not #12**	**216**
Date: 2019-07-10		
Number of results: 102 references		
*Cochrane reviews: -*		
*Other reviews: -*		
*Trials: 102*		
ID	**Search**	**Hits**
#1	hamstring or hamstrings:ti,ab,kw (Word variations have been searched)	1820
#2	MeSH descriptor: [Wounds and Injuries] this term only	2537
#3	MeSH descriptor: [Athletic Injuries] explode all trees	631
#4	MeSH descriptor: [Leg Injuries] explode all trees	4010
#5	MeSH descriptor: [Leg Injuries] this term only	192
#6	MeSH descriptor: [Sprains and Strains] this term only	390
#7	MeSH descriptor: [Tendon Injuries] this term only	236
#8	#2 or #3 or #4 or #5 or #6 or #7	7189
#9	injury or injuries or tear or tears or rupture or strain or strains:ti,ab,kw (Word variations have been searched)	77,212
#10	#8 or #9	76,921
#11	#1 and #10	737
#12	anterior cruciate ligament or “patellar tendon” or ACL or “posterior cruciate ligament”:ti (Word variations have been searched)	3057
#13	#11 not #12	327
#14	**#11 not #13 with Cochrane Library publication date Between May 2018 and Aug 2019**	**102**

**Table 8:** Modified Downs and Black checklist

Reporting	
1. Is the hypothesis/aim/objective of the study clearly described? 0-1p	
2. Are the main outcomes to be measured clearly described in the Introduction or Methods section? 0-1p	
3. Are the characteristics of the patients included in the study clearly described? 0-1p	
6. Are the main findings of the study clearly described? 0-1p	
7. Does the study provide estimates of the random variability in the data for the main outcomes? 0-1p	
9. Have the characteristics of patients lost to follow-up been described? 0-1p	
10. Have actual probability values been reported (e.g. 0.035 rather than < 0.05) for the main outcomes except where the probability value is less than 0.001? 0-1p	
External validity	
11. Were the subjects asked to participate in the study representative of the entire population from which they were recruited? 0-1p	
12. Were those subjects who were prepared to participate representative of the entire population from which they were recruited? 0-1p	
Internal validity – bias	
16. If any of the results of the study were based on “data dredging”, was this made clear? 0-1p	
17. In trials and cohort studies, do the analyses adjust for different lengths of follow-up of patients, or, in case-control studies, is the time period between the intervention and outcome the same for cases and controls? 0-1p	
18. Were the statistical tests used to assess the main outcomes appropriate? 0-1p	
20. Were the main outcome measures used accurate (valid and reliable)? 0-1p	
Internal validity – confounding	
25. Was there adequate adjustment for confounding in the analyses from which the main findings were drawn? 0-1p	
26. Were losses of patients to follow-up taken into account? 0-1p	
Power	
27. Did the study have sufficient power to detect a clinically important effect where the probability value for a difference being due to chance is less than 5%? 0-5p	

**Table 9:** Summary of included studies stratified by results and methods used to evaluate injury mechanism

Authors	Subjects (n)	Aim/purpose	Methods	^b^D&B	Results	Conclusion
Passive tension injuries						
Askling et al. [19]^a^	15	Investigate the injury mechanism, location and other factors related to acute, first-time hamstring injuries in dancers.	Interview, clinical and MRI examination.	11	Injury occurred while performing a slow-hip flexion with the knee extended in all cases. The location of injuries was close to the ischial tuberosity and most commonly affected the SM (87%), quadratus femoris (87%) and adductor magnus (33%). There were no significant findings in clinical or MRI examinations to determine return to preinjury level.	Stretching movements with simultaneous hip flexion and knee extension can cause a specific type of hamstring injury.
Askling et al. [31]^a^	30	Continued investigation of the injury location and recovery time for hamstring injuries in dancers.	Interview, clinical and MRI examination.	10	In all cases, injury occurred close to the ischial tuberosity while the hip was flexed and the knee extended, most commonly in the SM. 47% of the subjects ended their sports activity and there was no significant parameter during clinical or MRI examinations to predict time until return to sport.	Extensive hip flexion with the knee extended can cause a specific type of hamstring injury near the ischial tuberosity.
Sallay et al. [45]	12	Define the injury mechanism and present pathological changes, functional limitations and preventive measures in water skiers.	Interview, clinical examination. In five cases, MRI and in one CT scan.	8	The situation varied although injury occurred due to extensive hip flexion with an extended knee. The injuries were located proximal to the posterior thigh and time until return to sport varied from three months to 1.5 years.	Rapid stretching of the hamstrings can cause a hamstring injury.
Kinematic studies						
Hanley et al. [35]	17	Analyse the work done by the lower limb in world-class race walkers.	Race walking on a 45 m long track, with force plates to measure ground reaction forces, at competition speeds, captured at 100 Hz.	9	Most energy was generated by the extensors and flexors of the hip and during the late stance phase from the ankle plantarflexors. The knee flexors performed the most negative work and absorbed energy during the swing phase.	Injury is most likely to occur during the swing phase due to the negative work performed here which is increased by the straight knee during the first half of the stance phase.
Heiderscheit et al. [37]	1	Identify the time of injury in the gait cycle and the associated biomechanics of a hamstring injury.	Thirty-four reflective markers while running on a treadmill captured at 120 Hz. Toe markers were used to determine ground contact.	8	Based on the first signs of injury, 130 ms of the late swing phase was where the injury occurred. Moreover, during this phase, the biceps femoris reached peak musculotendon length.	The biceps femoris is probably injured during the late swing phase due to eccentric workload.
Fiorentino et al. [32]^a^	14	To create and validate a model of the BFlh from MRI-obtained information to predict local tissue strain during sprinting.	A model of the biceps femoris long head was made after measuring dimensions using an MRI camera. The model was validated and then used to perform a forward dynamic simulation of sprinting at different speeds.	8	By comparing in-vivo tissue strain from dynamic MRI experiments, the model used was shown to be working. Sprinting simulations showed the highest tissue strain in the BFlh at the proximal tendinous junctions which increased with increased sprinting speed.	The performed simulations showed non-uniform strain of the local fibres of the Bflh during the late swing phase which was predicted to increase with increased running speed.
Higashihara et al. [39]	8	To investigate differences in hamstring muscle kinematics during sprinting with different positions of the trunk	Thirty-four reflective markers captured at 200 Hz while the subjects ran two maximum-effort sprints, one with forward trunk lean and the other with an upright posture.	8	The forward trunk lean showed higher musculotendon length during the stance phase than upright running. Moreover, the late stance phase showed the highest positive musculotendon lengthening velocity with significantly higher values during the forward trunk lean.	Sprinting with a forward trunk lean causes the hamstrings to be more susceptible to injury during the stance phase.
Mann et al. [28]^a^	15	To help increase the knowledge of the kinematics during the ground phase of running.	Subjects were marked at anatomical landmarks and then had 40 m to reach maximum speed before being filmed at 150 frames/second. At least three trials/person.	8	During the stance phase, hip extensors performed concentric work from touchdown into the mid-support phase where activity shifted to the hip flexors which performed eccentric work through take-off. Muscles around the knee were dominated by flexors from touchdown to mid-support where dominance shifted to extensors, both performing eccentric work followed by concentric. At take-off, the flexors again performed eccentric work. Through the stance phase, plantar flexors were active and performed eccentric followed by concentric work.	Injury may occur because of the large forces working on the hamstrings when the foot touches the ground.
Schache et al. [46]	1	Compare the work performed by the different hip extensors and knee flexors during sprinting, as well as investigating asymmetries. Moreover, to compare the load on the hamstrings in different movements and before and after an injury.	Thirty-six reflective markers captured at 120 Hz while running at different speeds on a track containing force plates before suffering a hamstring injury on the 10th sprint.	8	During the terminal swing phase, the hamstring contributed to hip extension and knee flexion and peak force was shown to be greatest there while sprinting. After the hamstring injury occurred, the hamstring was unable to perform eccentric actions.	Because of the eccentric work performed during terminal swing, the hamstrings are most probably injured in this phase.
Schache et al. [47]	1	Investigate asymmetries before, the biomechanical response to and timing of an injury.	A previously injured athlete ran nine 30 m sprints with reflective markers mounted on him, while captured at 120 Hz, on a running track with two force plates before suffering a hamstring injury on the tenth sprint.	8	The first sign of injury was seen during the stance phase, but, due to neuromuscular latency, the calculated time of injury is prior to foot strike. Biomechanical asymmetries were seen in trials prior to the injury.	When sprinting, the hamstrings are most susceptible to injury during the terminal swing phase because of the eccentric work performed there.
Sun et al. [51]	8	Investigate hamstring kinematics and load in sprinting.	Isokinetic strength was measured before sprint trials. Fifty-seven reflective markers on anatomical landmarks. Captured at 300 Hz during three to four maximum-effort sprints on a track. GRF through force plates.	8	During both the initial stance and late swing phase, the hamstrings were subject to increased loading through forces working in opposite directions when the hip was extending and the knee flexing at the same time.	Sprinting or high-speed locomotion forces work on the hamstrings at the knee and hip during both the initial stance and the late swing phase which may cause an injury.
Thelen et al. [52]	14	Help understand the hamstring injury mechanism by investigating the work of the hamstrings in sprinting.	Forty-seven reflective markers on anatomical landmarks. Running on a treadmill at different speeds recorded at 200 Hz.	7	The peak length of the hamstrings was measured during the late swing phase with the biceps femoris being significantly higher and occurring later than the other muscles in the hamstring muscle group. However, no significant difference was found depending on sprinting speeds.	The greatest peak length is found in the biceps femoris during the late swing while sprinting, which is why hamstring injuries are most likely to occur there.
Wan et al. [53]	20	To investigate whether hamstring flexibility relates to peak hamstring muscle strain during sprinting.	Flexibility was measured with a passive straight leg raise after a sufficient warm-up. Sprinting kinematics were measured with reflective markers on anatomical landmarks and filmed at 200 frames/second while performing 20-25 m sprints. Bilateral isokinetic strength tests were also performed.	9	Peak muscle strain of all the hamstring muscles were recorded during the late swing phase and correlated negatively to hamstring flexibility. No gender differences were recorded. The strain in the BFlh and ST was higher than in the SM.	In sprinting, the hamstrings exhibit injury potential during the late swing phase.
Kinematic studies with EMG analyses						
Chumanov et al. [34]	12	Compare the hamstring mechanics in the swing and stance phase during sprinting.	Forty-five reflective markers along with surface electrodes, the latter placed on seven muscles of the lower right extremity, were mounted on the subjects before running on a treadmill at different speeds.	9	Eccentric contraction was measured in the hamstring during the swing phase before switching to concentric contraction during late swing which lasted through the stance phase. Increased sprinting speed meant an increased load for the biceps femoris.	The late swing phase is more injury prone than the stance phase during sprinting.
Hanley et al. [36]	20	To investigate the lower extremity during race walking.	Race walking on a 45 m track at competitive speed while filmed at 100 Hz and walking over force plates with surface electrodes on seven muscles of the lower right extremity.	9	Hip extensors during late swing and early stance along with ankle plantarflexors during late stance were the most important in producing energy. Great negative work was seen by knee flexors during the swing phase.	The risk of injury to the hamstrings is highest during the swing phase, due to the negative work performed there.
Higashihara et al. [38]	13	Investigate the hamstring injury mechanism by analysing peak musculotendon length and EMG activity during sprinting.	Forty m acceleration was allowed on a synthetic track. Thirty-four reflective markers captured at 200 Hz. Surface electrodes on the muscle bellies of BFlh and ST with one on the fibular head for reference.	8	For the biceps femoris, the maximum length and peak EMG activity occurred at the same time during the late swing phase. For the ST, the highest EMG activity was measured before it reached its maximum length.	The hamstrings are most likely to be injured during the late swing phase while sprinting.
Montgomery III et al. [33]^a^	30	Investigate EMG activity of muscles around the hip and knee while running at different speeds.	Needle electrodes were placed in three to eight muscles before performing runs at self-determined speeds in front of a high-speed camera.	8	The quadriceps had its major activity during the early stance as knee extensors, hamstrings were active in both knee flexion and hip extension during two to three periods of the gait cycle. Hip flexion was mainly performed by the rectus femoris during stance and iliacus during early-middle swing.	The hamstrings are injured during the swing phase due to eccentric contraction, but the different muscles of the hamstring muscle group are not susceptible at exactly the same time.
Ono et al. [41]	12	Investigate when a hamstring injury occurs by estimating tensile force during sprinting.	Reflective markers, high-speed cameras, force plates and surface electrodes were used to sample data from the subjects while running at maximum speeds on a 50 m track. A maximum voluntary contraction was used as an EMG reference.	8	Peak values for strain of the hamstring were shown during late swing with the highest values in the ST. The BFlh peak EMG activity took place directly after the foot touched the ground.	The BFlh is most likely to be injured during the early stance phase.
Padulo et al. [42]	12	Investigate the hamstring during movements with different types of muscle contraction.	Biceps femoris EMG activity was measured by surface electrodes and subjects were filmed with a high-speed camera while performing a counter-movement jump, squat jump and landing from a 45 cm high box. A maximum voluntary contraction was used as a reference value for the EMG.	8	When comparing a counter-movement jump with a squat jump and the braking phase of a landing, the biceps femoris showed lower activation, in both the concentric and eccentric phases of the counter-movement jump.	A pure eccentric or concentric movement gives rise to higher neuromuscular activity than a stretch-shortening exercise.
Prior et al. [43]	22	Investigate how trunk and pelvis positions affect the muscles of the thigh and hip while standing on one leg.	Markers, high-speed cameras and surface EMG of eight different muscles on both body halves was measured with the subject standing on one leg in different posture and pelvic positions.	14	When comparing anterior with posterior trunk sway during a one-legged stance, the muscles situated in a posterior position in the sagittal plane increased their activity as the anterior muscles decreased their activity. When swaying to the opposite side compared with the same side as the stance leg, the lateral hip abductor activity increased. A lateral drop of the pelvis, compared with a rise, reduced hip abductor activity while the hamstring, adductor longus and vastus lateralis increased their activity.	Trunk and pelvic positions affect the activation of the muscles around the hip and may increase the risk of injury.
Ruan et al. [44]	12	Investigate the effect of static stretching on hamstring injury risk.	Surface EMG, reflective markers, high-speed cameras and force plates collected data to compare parameters before and after a passive static stretch of the hamstrings.	8	The static stretch increased maximum BFlh length without affecting knee flexion torque. It also reduced peak GRF during the early stance phase and hamstring activation during the late swing phase.	The effects of static stretching during both the late swing and early stance phase may help reduce hamstring injuries.
Schache et al. [48]	7	Investigate the loading of the different muscles of the hamstring muscle group during sprinting.	A 110 m running track with embedded force plates was used. Subjects ran maximum sprints with 50 reflective markers captured at 250 Hz while having surface electrodes mounted on the hamstrings with a reference one on the tibial shaft.	8	All hamstring muscles reached their peak values regarding strain and force produced during terminal swing where they also performed negative work. The highest strain was found in the BFlh, the greatest lengthening velocity was found in ST and the highest force was found to be produced by SM which also performed the most work, both negative and positive.	The hamstrings are most likely to be injured during the terminal swing phase.
Yu et al. [54]	20	Investigate hamstring kinematics and activation to obtain knowledge of the hamstring injury mechanism.	Surface electrodes were placed on the dominant semimembranosus and biceps femoris along with bilateral reflective markers before maximum sprints were performed on an indoor track.	9	During both the late stance and late swing phase, the hamstring contracted eccentrically. The eccentric contraction speed showed a significantly higher peak value during the late swing. However, the peak value musculotendon lengths were significantly higher during the late stance.	The hamstrings may suffer an injury because of an eccentric contraction during both the late swing and late stance phases.
Strength-related injuries						
Jones et al. [40]	20	Investigate how fatigue affects muscle strength in football players from Africa.	Athletes performed a maximum concentric knee extension and maximum eccentric knee flexion before, during and after a workout protocol that simulates the fatigue of a football game.	9	The workout protocol generated significantly lower concentric quadriceps and eccentric hamstring strength. Moreover, the ratio between them (eccentric hamstring:concentric quadriceps) decreased significantly.	Fatigue-induced eccentric strength deficiency may be the reason for hamstring injuries in football players.
Schuermans et al. [49]	54	Investigate how synergistic work by muscles of the hamstring muscle group affects hamstring injuries.	Twenty-seven uninjured and 27 previously injured athletes underwent an MRI scan of the hamstring before and after an eccentric exercise.	10	The formerly injured group had a significantly more symmetrical muscle recruitment which corresponds to less economic muscle activation. Moreover, the group that had previously suffered an injury showed lower eccentric strength endurance.	Hamstring injuries may be related to the synergistic recruitment of the different muscles.
Schuermans et al. [50]	54	To identify the risk of future hamstring injuries with the help of mfMRI.	All players underwent an mfMRI scan before and after an eccentric hamstring exercise. Hamstring injuries were then registered for the following 1.5 seasons.	11	A first-time hamstring injury was associated with a high metabolic response and a proportionally higher biceps femoris contribution. A re-injury was associated with lower eccentric hamstring endurance.	An increase in the metabolic activity of the BF is predictive of sustaining an index hamstring injury. Concerning re-injuries, lower eccentric endurance capacity is able to predict a recurrent hamstring injury.

^a^Identified through reference lists, ^b^Downs & Black Checklist Score, *BF* Biceps femoris, *BFlh* Biceps femoris long head, *cm* Centimetre, *CT* Computed tomography, *EMG* Electromyography, *GRF* Ground reaction force, *Hz* Hertz, *M* Metre, *MRI* Magnetic resonance imaging, *mfMRI* Muscle functional magnetic resonance imaging, *Ms*. Milliseconds, *ST* Semitendinosus, *SM* Semimembranosus

## Abbreviations

BFlh: Long head of the biceps femoris
SM: Semimembranosus
EMG: Electromyography
MRI: Magnetic resonance imaging
mfMRI: Muscle functional magnetic resonance imaging
ST: Semitendinosus
N/A: Not applicable
GRF: Ground reaction force
